# Caregiver and provider perspectives on the needs of families waiting for trauma therapy

**DOI:** 10.1002/jts.23191

**Published:** 2025-07-23

**Authors:** Caitlin Rancher, Owen Winters, Angela D. Moreland, Daniel W. Smith

**Affiliations:** ^1^ National Crime Victims Research Treatment Center, Department of Psychiatry and Behavioral Sciences Medical University of South Carolina Charleston South Carolina USA

## Abstract

Children's exposure to trauma is a ubiquitous stressor associated with severe adjustment problems. Although mental health services can effectively reduce this distress, families often spend months waiting for treatment. Beyond timely access to care, the needs and concerns of families on the waitlist for trauma‐focused treatment are unknown. In the present study, we addressed this gap by conducting interviews with key participant groups: adult caregivers with a child on the waitlist for trauma‐focused services (*n* = 16) and providers of trauma‐focused treatment (*n* = 17). Participants completed semistructured interviews on the needs and concerns of families on the waitlist for trauma‐focused mental health services for their child. Interview transcripts were analyzed using a combination of deductive and inductive thematic analysis. Consolidated thematic coding yielded four themes related to waitlist concerns: waitlist duration, worsening symptoms, family not feeling supported, and safety. Six additional themes emerged related to waitlist needs: parenting services, psychoeducation on trauma, check‐ins on well‐being and status, case management, financial resources, and referrals to other services. The results suggest that both caregivers and providers are largely aligned in their perspectives of the needs and concerns of families on the waitlist for trauma‐focused treatment. Several of the identified needs could be addressed by low‐intensity interventions delivered by paraprofessionals. The accessibility and appropriateness of providing support to families on the waitlist for treatment should be explored.

Childhood exposure to traumatic events, such as abuse, violence, or sudden loss, is a prevalent public health concern, often resulting in heightened adjustment problems, including trauma symptoms, depression, anxiety, and substance misuse (Alisic et al., 2014; Kihas et al., 2024; Stoltenborgh et al., 2015). Fortunately, many empirically supported treatments have been found to effectively reduce distress resulting from trauma exposure (Diehle et al., 2014; Jericho et al., 2022). These treatments, however, tend to be lengthy and require extensive provider training to deliver with fidelity; as a result, families often experience long wait times before beginning treatment (Jouriles et al., 2024; Peipert et al., 2022; Rancher et al., in press).

In recent years, families have been spending even more time waiting for treatment due to a combination of provider shortages and a heightened demand for mental health services (Azzopardi et al., 2022). Lengthy wait times, often 2–5 months, have been reported among families seeking treatment to address children's exposure to trauma (Jouriles et al., 2025; Rancher et al., in press). This is a critical problem, as time spent waiting for mental health treatment has been correlated with higher levels of psychological distress, higher rates of premature attrition, lower engagement in treatment, and discouragement from even attempting to access treatment (Krendl & Lorenzo‐Luaces, 2022; Punton et al., 2022; Rancher et al., in press).

Despite these well‐established consequences, little is known about the needs and concerns of families while they are waiting for treatment. Beyond the provision of more timely access to care, it is unclear what strategies or interventions would be most helpful to mitigate some of the consequences of lengthy waitlists. For example, low‐intensity treatments have been suggested as a potential solution to reduce distress among individuals waiting for treatment (Levin et al., 2022); however, the actual needs or best‐suited interventions for families are unclear. Indeed, recent qualitative analyses of the perspectives of mental health providers suggest that the utility of low‐intensity treatments for families on the waitlist for mental health services may be limited by a lack of understanding of patients’ specific needs (Peipert et al., 2022). This information is critical to understand, as tailoring interventions to address specific needs and concerns has been found to result in more treatment engagement and improved clinical outcomes (Benjamin et al., 2025; Rathod et al., 2018).

To best address this public health gap, more insight is needed into the actual needs and concerns of families who are waiting for trauma‐focused treatment. To increase knowledge in this area, we conducted qualitative interviews with two groups: caregivers of a child on the waitlist for trauma services and providers of trauma‐focused treatment for children. Caregivers can provide critical insight into the lived experience of their family while on the waitlist. Providers have the benefit of working with multiple families and can speak to broader patterns of needs among families on waitlists for treatment. Perspectives from both key participant groups are essential to best understanding the needs and concerns of families waiting for trauma therapy.

## METHOD

### Participants and procedure

Data collection took place at a training clinic providing trauma‐focused mental health services in the southern United States. This clinic is located within an academic medical center and accepts patients with federal coverage (Medicaid, Medicare), state coverage (crime victim's compensation), and private insurance, as well as those who self‐pay. Key participant groups were identified as adult caregivers with a child on the waitlist for trauma‐focused services (*n* = 16) and providers of trauma‐focused treatment (*n* = 17). As part of routine services at this clinic, all clients are asked whether they are willing to be contacted for volunteer research opportunities. Caregivers who provided permission were contacted by phone and invited to participate. Caregivers were informed that their decision to participate would not affect their status on the waitlist nor would it be included in their clinic treatment records. Individuals were eligible for inclusion if they were (a) an adult aged 18 years or older and (b) the caregiver to a child between 7 and 12 years of age who was (c) currently on the waitlist for trauma‐focused mental health services. Caregivers were invited to complete interviews in either English or Spanish.

This study was advertised on internal listservs sent to staff via email and presented at provider team meetings. Providers were informed their participation would not affect any performance evaluations or be included in their employment records and told that supervisors would not be informed of who participated in the study. Providers were eligible for inclusion if they were (a) an adult aged 18 years or older and (b) currently employed or receiving training at the trauma‐focused clinic. All participants provided written informed consent, completed a self‐report demographics questionnaire, and completed an individual qualitative interview on the needs and concerns of families waiting for trauma treatment (e.g., “What are some of the needs of caregivers and families on the waitlist for trauma‐focused mental health services for their child?”). Caregivers and providers were asked the same interview questions. Interviews were conducted by a research consultant with no prior relationship with the clinic, caregivers, or providers. All methods were carried out in accordance with the Declaration of Helsinki. All experimental protocols were approved by the Institutional Review Board at the Medical University of South Carolina.

Demographic characteristics are reported in Table 1. All caregivers identified as the child's mother (87.5% birth mother, 12.5% adoptive mother). Most caregivers (75.0%) had been referred for trauma‐focused services within the past 3 months; families had been on the waitlist for an average of 86 days (*SD* = 133.51). Providers represented diverse positions, including trainees (predoctoral clinical psychology interns or postdoctoral fellows; 23.5%), staff clinicians (41.2%), case managers (11.8%), and faculty or supervisors (23.5%).

**TABLE 1:** Demographic characteristics of the sample

Variable	Caregivers		Providers	
	(*n* = 16)		(*n* = 17)	
	*M*	*SD*	*M*	*SD*
Age (years)	41.88	8.39	34.65	8.35

	*n*	%	*n*	%
Language				
Spanish	6	37.5	0	0.0
English	10	62.5	17	100.0
Gender				
Female	16	100.0	15	88.2
Male	0	0.0	2	11.8
Race				
White/European American	6	37.5	11	64.7
Black/African American	4	25.0	3	17.6
Middle Eastern or North African	0	0.0	1	5.9
Multiracial	0	0.0	1	5.9
Other	6	37.5	1	5.9
Ethnicity				
Not Hispanic/Latinx	10	62.5	15	88.2
Hispanic/Latinx	6	37.5	2	11.8
Educational attainment				
Some high school	3	18.8	0	0.0
High school graduate	3	18.8	0	0.0
Some college or technical school	3	18.8	0	0.0
College graduate	7	43.8	17	100.0

### Data analysis

Interviews were audio recorded and transcribed verbatim to enable qualitative analyses. Spanish‐language transcripts were translated to English by a certified translator prior to analysis. Transcripts were analyzed using a combination of deductive and inductive thematic analysis, where specific themes were drawn from the interview guide and combined with themes that emerged organically from the data (Braun & Clarke, 2006). Two independent coders first analyzed the data to generate initial codes. Each coder then read through the data, and the coders worked in consensus to generate a codebook that captured salient themes. Once the codebook was established, one of the original coders and a new independent coder each applied the codes to the entire dataset. Interrater reliability was found to be in the substantial agreement range, κ = .75. Discrepancies were discussed in group meetings with three authors and negotiated to achieve consensus on final codes.

Original thematic coding yielded some overlap across themes. For example, two themes related to waitlist concerns—“symptoms not getting addressed” and “symptoms worsening”—captured extremely similar content and, thus, were combined into a single theme of “worsening symptoms.” Similarly, some themes related to waitlist needs (e.g., “needs assessment” and “case management”) coded nearly identical content and were consolidated into a single theme (“case management”). The codebook and deidentified transcripts are available from the first author upon request.

## RESULTS

Consolidated thematic coding yielded four themes related to waitlist concerns: waitlist duration, worsening symptoms, family not feeling supported, and safety. Themes and exemplar quotes related to waitlist concerns are presented in Table 2.

**TABLE 2:** Themes related to waitlist concerns

	Frequency				Example quote
	Caregiver		Provider		
Theme	*n*	%	*n*	%	
Waitlist duration	5	31.3	6	35.3	“Hard as a parent to explain that to your child, like help is coming, but it's just gonna be a while.” (Caregiver)
Worsening symptoms	12	75.0	12	70.6	“The risk of something more dangerous happening to the caregiver or the child, during that time because they don't have skills or resources yet to manage it on their own.” (Provider)
Family not feeling supported	8	50.0	9	52.9	“I worry about how much longer am I going to have to handle this on my own.” (Caregiver)
Safety	4	25.0	6	35.3	“Maybe they're still in contact with the perpetrator…maybe the child is engaging in risky behaviors.” (Provider)

### Waitlist concerns

#### Waitlist duration

Both groups of participants were concerned about the duration of time families spent on the waitlist. This theme was mentioned by approximately one third of caregivers (31.3%) and providers (35.3%). Caregivers explained how the time spent waiting can be challenging for their child to understand. As one caregiver noted:

The biggest, you know, concern as a parent, you don't want to see your kids struggle. And I know the mental health crisis is big for adults and for children, so services are hard to find for both. So, it's really tough as a parent to watch your kid kind of go through that, and then they don't understand…they don't understand the process of waiting. You know, they don't understand, like, hey, what does it mean to wait 6 months for somebody to help me? Like, they don't understand that concept. So, kind of hard as a parent to explain that to your child, like, help is coming, but it's just gonna be a while.

Providers explained how families often have spent a considerable amount of time waiting for relief from symptoms even before they are placed on the clinic waitlist. As one provider noted, it can “take a while for them to get to that point of reaching out…and then they have to wait on the waitlist.” As another provider remarked, “I think about it like, if I have four kids, if they needed to get into services because they were pretty distressed, waiting several months is unacceptable, right?”

#### Worsening symptoms

The most frequently discussed concern across both caregivers (75.0%) and providers (70.6%) was that the child's symptoms would get worse the longer the family spent waiting for treatment. As one caregiver explained, “It just scared me a lot because I just felt it was getting worse. The situation for him was getting worse, and it wasn't getting resolved.” For caregivers, this concern extended beyond their child's own mental health to the potential risk to others, as one caregiver stated:

He's going to get to the point where he's real bad because now, like sometimes he gets where, he[’s] like, bullying? The bullying type? Like, just wanting to hit people's kids. And I don't like that. He might get to the point where he is really acting out the wrong way and really just hurt somebody's child.

Providers similarly expressed concerns about worsening symptoms. As one provider commented, “The longer the time period goes on, sometimes the worse things could get.”

#### Family not feeling supported

Nearly half of the interviewed caregivers (50.0%) and providers (52.9%) expressed concerns that lengthy waitlists can lead to families feeling discouraged and unsupported. Caregivers noted that not feeling supported while waiting for treatment caused additional distress. One caregiver said:

Sitting in the dark, yeah, when you're sitting in the dark, it's like, it's almost as if…you're stuck there, even for someone with a little bit of anxiety. That's a huge thing. So, for someone who's experienced trauma, sitting in the dark without the reassurance….It can be an even heavier load.

Alternatively, for providers, this concern was related to lower levels of family engagement. One provider explained, “Sometimes we just have trouble getting in touch with people because they're like, by the time we come around, they're like, F that, you know?”

#### Safety

Approximately one quarter of caregivers (25.0%) and one third of providers (35.3%) described feeling concerned about the child's safety while the family was waiting for services. Some caregivers explained they were concerned about their child's safety in terms of suicidal ideation or self‐harm. As one caregiver described, her concern for the safety of her son was “that he may become suicidal…that he can come to think or have suicidal thoughts because he believes that nobody cares about him, or because of bullying at school, or because of how his friends at school treat him.” Both providers and caregivers expressed safety concerns related to ongoing contact with the alleged perpetrator of maltreatment or abuse. As one provider noted,

You know, if there's excessive corporal punishment, or if kids are at all unsafe in the home, of course, we don't always…we're not able to identify that just from phone screens or whatever—not all the time—but that would be a concern.

### Waitlist needs

An additional six themes emerged related to waitlist needs: parenting services, psychoeducation on trauma, check‐ins on well‐being and status, case management, financial resources, and referrals to other services. Themes and exemplar quotes related to waitlist needs are presented in Table 3.

**TABLE 3:** Themes related to waitlist needs

	Frequency				
	Caregiver		Provider		
Theme	*n*	%	*n*	%	Example
Parenting services	13	81.3	17	100.0	“You know, as a parent, you don't know what to do when your kid is struggling, especially when you're struggling yourself.” (Caregiver)
Psychoeducation on trauma	4	25.0	13	76.5	“I see a lot of needs in understanding, like what trauma might look like.” (Provider)
Check‐ins	5	31.3	7	41.2	“It might be helpful for parents to be able to reach out to someone on the waitlist, periodically have a contact for updates.” (Provider)
Case management	6	37.5	12	70.6	“Guide us through, like, where do we go from here? And how do we handle this in court?” (Caregiver)
Financial resources	4	25.0	9	52.9	“I also had financial needs in many ways because sometimes mental health is affected by everything that is circumstantial.” (Caregiver)
Referrals to other services	8	50.0	9	52.9	“For some of the events that children have come in for seeking services, the parent has also either experienced that event as well, or they're not doing as well, and also want their own services.” (Provider)

#### Parenting services

The waitlist need both caregivers (81.3%) and providers (100.0%) identified most frequently was support for parenting skills. Caregivers expressed a desire for guidance on parenting. As one caregiver noted, “I would say more specific training or more maybe one‐on‐one counseling with, I don't know, how to take care of a child or what kind of things that we could do.” In general, caregivers were able to identify that, as a parent, they were struggling and did not know how to best support their child. As one caregiver stated:

I didn't know who to go to, what to do, you know?…As a parent, you know, I[’ve] just been trying to do parenting coping skills, you know, kept assuring her, ‘this is OK,’ you know…there's many, many, many, many nights that I cry, and I'm like, ‘What am I going to do? What can I do for her?’ I'm hurting for her.

Providers identified a range of possible options to support parenting skills, including general concepts, such as “interim parenting‐specific support,” as well as concrete parenting skills. One provider suggested that caregivers need “ways to manage behavior problems or increasing positive time and just how to respond if your child wants to talk about it or not.”

#### Psychoeducation on trauma

One quarter of caregivers (25.0%) and the majority of providers (76.5%) identified psychoeducation on trauma symptoms as a need for families on the waitlist. Interviewed caregivers were focused on a desire for information from trusted resources. As one caregiver explained, “I need information. I feel like I don't…I mean, I could Google it, but I'm like, I don't trust Google. I want to…I'd like to get professional advice.” Providers explained how this information can be helpful in preparing families for later trauma‐focused treatment. As one provider noted:

Just from, like, clinical experience, a lot of our caregivers have limited emotional vocabulary themselves, so I think that could be really helpful to start expanding that skill early on. I could definitely see that feeding into having some foundation to build upon in therapy.

Providers also described how providing psychoeducation on common responses to trauma can help provide some reassurance for caregivers and instill hope about the effectiveness of treatment. As one provider stated:

Start with psychoeducation about trauma and common responses to trauma, some validating support for caregivers that it's hard, you know, to support a child with trauma, and, you know, kind of validating, like, these things are normal. A lot of times we hear caregivers kind of come in and they're like, ‘Is my child broken as a child, you know, a sociopath?’ And all of these things that are like, ‘No, no, no, this is just common trauma responses, and the good news is, you know, we have treatments for that.’ And, so, kind of like highlighting that there's good news, that there is a lot of hope, and they know they're on the waitlist right now, but once they get in, there's so much hope with this treatment.

#### Check‐ins on well‐being and waitlist status

Both caregivers and providers identified the need for check‐ins for families on the waitlist; approximately one third of caregivers (31.3%) and nearly half of providers (41.2%) mentioned this theme. Caregivers identified the need for check‐ins on the status of the waitlist as well as the need for check‐ins on their family's well‐being. One caregiver suggested, “Like, ‘Hey, you know, we still have you on the waitlist’ or ‘Hey, I hope you're doing OK.’” Other caregivers explained how these check‐ins would help them feel more supported by the clinic. As one caregiver commented:

Communicate with me. Don't just put me on a waiting list and then forget about me until my name comes up. You know, I'm not a number, I'm a person, you know? I am a person with, you know, situations that I need to be addressed and helped with—that's why I'm reaching out to you. I guess that's the thing, too, that people fall short on, is the communication and the keeping in touch. ‘Hey, I'm just, you know, reaching out to you to let you know we haven't forgot[ten] about you. I understand, you know, the wait is frustrating, this and that, but you know, I just want to let you know that we are here. We are doing what we can.’ You know? Just to try help reassure that parent or that caregiver.

Alternatively, for providers, the check‐ins were described as primarily focused on sharing updates on the status of the waitlist and how long the family might expect to wait for a provider. As one provider described, “I think it would be nice to have more regular check‐ins, like, you know, for a 6‐month waitlist. Are we checking in every couple of weeks to a month?”

#### Case management

Over one third of caregivers (37.5%) and most providers (70.6%) identified that families on the waitlist need case management support. For caregivers, the need for case management was more related to knowing how to navigate the health care and court systems. As one caregiver explained, “I think a lot of people that are probably victims of crime, maybe they're not educated in the health care system, so, you know, anything to help those poor kids in that situation, I think would be great.” On the other hand, providers noted that many families are experiencing stressors beyond the traumatic event itself, which can exacerbate distress and present barriers to later treatment attendance. As one provider said, “A lot of what I hear is, like, they don't know where to start in the event of a crisis. Where do they go for resources? Rent's past due, utilities [are] past due, is there assistance for that?” Another provider described how this might involve conducting a basic needs assessment for each family to inform case management services: “An assessment for what basic needs they've got, you know, food, clothing, shelter…connecting them with services that they need. A lot of that can be very impactful and help reduce their distress even if they're on the waitlist.”

#### Financial resources

Approximately one quarter of caregivers (25.0%) and half of providers (52.9%) identified financial resources as a need for families on the waitlist. Caregivers identified the need for financial resources to support paying for mental health services. One caregiver expressed this frustration with their insurance coverage:

My insurance is…my husband's a teacher, and it's state insurance, and I just can't believe that it's not accepted. So that's just another frustration too…the financial aspect of it and, you know, that it's not covered by that insurance…like, it should be.

For providers, the need for financial resources was related to external stressors among low‐resource families. According to one provider, “Thinking about the hierarchy of needs, sometimes we have kids [who] are even food insecure, you know, like, not even talking about mental health, but food or housing insecure. And sometimes families just need those tangible support resources.” Providers discussed the importance of distributing financial resources so families can focus on addressing mental health concerns.

#### Referrals to other services

Approximately half of both caregivers (50.0%) and providers (52.9%) commented on the need to provide families with referrals to other services. Caregivers explained how they need referrals for other services given their urgency to help relieve their child's distress. As one caregiver said, “We need to do *something*. If you can't help me, then point me somewhere else. But I'm not just letting it go! Something is wrong! Something is going on! We need to do something!” For both groups, these additional referrals also included mental health referrals for the caregivers themselves. As one provider described:

Usually, the child [who's] on the waitlist isn't the only one in the family [who] needs mental health services. A lot of times, caregivers themselves have either been traumatized or are having secondary traumatic stress from their child's victimization or just have their own stuff going on. It might not necessarily be trauma‐focused counseling that they need, but just general counseling support.

## DISCUSSION

In this study, we interviewed caregivers and providers about the needs and concerns of families waiting for their child to receive trauma‐focused treatment. Thematic analyses revealed that both groups of participants were concerned about the waitlist duration, worsening symptoms, families not feeling supported, and child safety. Caregivers and providers also identified the needs of families on the waitlist, including parenting services, psychoeducation on trauma, check‐ins on well‐being and status, case management, financial resources, and referrals to other services. Families are spending months on mental health waitlists for their child to receive trauma‐focused treatment (Jouriles et al., 2025; Rancher et al., in press). Understanding the types of interventions or resources that would best support families during this waiting time is critical for providers and policymakers.

The waitlist concern that both caregivers (75.0%) and providers (70.6%) endorsed most frequently was worsening symptoms. There is significant concern that the longer a family spends waiting for treatment, the more distress the child will experience. It is possible that an increase in distress while waiting for treatment partially explains previous findings that longer wait times for mental health treatment are associated with worse treatment outcomes (van Dijk et al., 2023). The development of distressing, impairing symptoms is often the catalyst for caregivers to seek trauma‐focused treatment for their child in the first place. Unfortunately, children's trauma‐related distress rarely resolves without intervention (Scheeringa & Zeanah, 2008) and, when left untreated, can be associated with long‐term disruptions in physical health, emotion regulation, relationships, and psychological well‐being (Dye, 2018; McFarlane, 2010; Rooks et al., 2015). Worsening symptoms and the potential for long‐term functional impairment is a major concern for both caregivers and providers. This concern highlights the critical need to offer support to families waiting for intensive treatment.

Fortunately, both groups of participants offered suggestions on ways to address the needs of families waiting for trauma‐focused treatment. The majority of caregivers (81.3%) and every provider (100.0%) identified parenting services as a waitlist need. Caregivers play a pivotal role in helping their child recover from trauma by providing emotional support and facilitating access to mental health treatment (Brown et al., 2020; Gower et al., 2020). Unfortunately, many caregivers lack the skills or self‐efficacy to effectively support their child following a traumatic event (Theimer et al., 2020; Williamson et al., 2017). The present results suggest that providing some type of parenting support to families on the waitlist would be helpful. Brief parenting programs that focus on teaching specific positive parenting skills to enhance the parent–child relationship may be particularly apt. These programs have been found to effectively enhance parenting skills in as few as three or four sessions, predict engagement in later trauma‐focused treatment, and can be delivered by paraprofessionals with high levels of fidelity (Rancher et al., 2023). Recruiting paraprofessional providers to deliver these brief, low‐intensity programs may be one way to extend the reach of evidence‐based care, and meet the needs of families waiting for treatment, in a more cost‐effective manner than recruiting and retaining licensed clinicians. Still, future research is needed on the acceptability and feasibility of delivering a brief parenting program to families on the waitlist for trauma services.

Although caregivers and providers were largely aligned in their discussion of waitlist needs, one major area of discrepancy emerged in the dissemination of psychoeducation. Only one quarter of caregivers (25.0%) described needing psychoeducation on trauma compared to the majority of providers (76.5%), who reported that families needed this information. The provision of psychoeducation is often the first step of many intensive empirically supported treatments (e.g., trauma‐focused cognitive behavior therapy; Cohen et al., 2016). Providers explained how this could be a helpful foundation for future trauma treatment—offering information on common trauma symptoms and the focus of therapy may help families be more prepared to start treatment. It is worth considering that this is a concrete, didactic skill that can be delivered by unlicensed paraprofessionals before families receive more intensive treatment. It also seems possible that caregivers might be receptive to receiving information even if they do not independently identify psychoeducation as a need. That is, they might not know to ask for this information or that this is a critical component in treatment and recovery. Across multiple cluster‐randomized trials, preparing consumers to be active participants in their care—providing explicit information about common symptoms and offering the opportunity to ask questions about treatment decisions—has been identified as an effective implementation strategy that increases treatment engagement and adherence to best practices (see Hero et al., 2023, for a review). This strategy, called “caregiver patient activation,” results in caregivers requesting more information about evidence‐based treatment, which, in turn, can enhance provider beliefs in the acceptability of an intervention and increase the number of families who receive care (Coulter, 2012; Krist et al., 2017). To our knowledge, caregiver patient activation is an underutilized strategy for engaging families waiting for trauma treatment. In general, future research may consider exploring the benefits of providing quality, accurate psychoeducation on trauma to families on the waitlists for services.

One clinical implication of the present study is the need for more frequent contact or check‐ins with families on the waitlist. This theme, echoed by substantial numbers of caregivers (31.3%) and providers (41.2%), highlights the importance of making sure families receive some ongoing contact while waiting for treatment. Given estimates that less than half (46%) of children who are referred for trauma‐focused services begin treatment (National Children's Alliance, 2019), check‐ins might be one way to gauge ongoing interest in services. These contacts, which could simply include updates on the current waitlist duration, are another example of a brief intervention that could be staffed by paraprofessionals to dramatically enhance the quality of care provided by trauma‐focused mental health clinics. It may also be that these check‐ins are opportunities to address case management and financial resource needs and assess for the potential worsening of symptoms. Many families may benefit from the provision of information on how to access financial resources or donated goods. Survivors of crime are also sometimes eligible to receive financial compensation. Crime victims’ compensation programs can provide reimbursement to families to cover crime‐related expenses, including medical costs, mental health services, and lost wages. Providers at trauma‐focused clinics may be uniquely positioned to offer guidance on these resources and can use the time families spend waiting for treatment to offer instrumental support.

There are several limitations to the present research. Foremost, the caregivers and providers interviewed in the present study were drawn from a single trauma‐focused training clinic in the southern United States. The experiences of families and providers at this clinic may be limited in their generalizability to other clinical settings, such as community mental health clinics, child advocacy centers, or private practices. The feasibility of providing parenting interventions, psychoeducation, or check‐ins may differ across contexts in terms of available paraprofessionals, financial resources, and competing clinic priorities; for example, child advocacy centers frequently have more system‐level resources, such as family advocates who could deliver low‐intensity services, compared to community mental health clinics. That said, brief parenting programs and patient activation are strategies that have been found to enhance treatment engagement across diverse clinical contexts (Hero et al., 2023; Rancher et al., 2023), and future implementation research is warranted to explore the dissemination of these strategies. Caregiver perspectives in the current study were also drawn from individuals who indicated they were willing to participate in volunteer research opportunities and, thus, may differ from those who are unwilling to participate in research. Finally, the qualitative interview guide was designed to assess the needs of families on the waitlist for trauma‐focused services using open‐ended questions; however, additional concerns and needs that were not addressed likely exist in this sample. Future research using longer, established measures should build on these findings.

This study addresses the public health gap regarding understanding the actual needs and concerns of families waiting for trauma‐focused treatment. We observed significant alignment across the perspectives of caregivers with a child on the waitlist and providers of trauma treatment. Both groups of participants expressed concern over the duration of time spent waiting for treatment and the possibility that the child's symptoms would worsen while waiting for treatment. Fortunately, many of the identified needs could be addressed by low‐intensity interventions, many of which could be staffed by paraprofessionals. Leveraging this workforce may be one way to decrease the burden on licensed providers and ensure more timely delivery of supportive services to families. As one caregiver noted, there is a pressing urgency to “do *something*” for families who are spending months waiting for trauma treatment. The present findings highlight the need to provide support to families on the waitlist, and future research should target the accessibility and appropriateness of providing support through brief, low‐intensity interventions.

## AUTHOR NOTE

Caitlin Rancher was supported by the Eunice Kennedy Shriver National Institute of Child Health and Human Development (K99HD111677).

## OPEN PRACTICES STATEMENT

This study was not formally preregistered. Neither the data nor the materials have been made available on a permanent third‐party archive; requests for the data or materials can be sent via email to the lead author, Caitlin Rancher, at rancher@musc.edu
